# Deep Phenotyping of CD11c^+^ B Cells in Systemic Autoimmunity and Controls

**DOI:** 10.3389/fimmu.2021.635615

**Published:** 2021-03-12

**Authors:** Hector Rincon-Arevalo, Annika Wiedemann, Ana-Luisa Stefanski, Marie Lettau, Franziska Szelinski, Sebastian Fuchs, Andreas Philipp Frei, Malte Steinberg, Tony Kam-Thong, Klas Hatje, Baerbel Keller, Klaus Warnatz, Andreas Radbruch, Andreia C. Lino, Eva Schrezenmeier, Thomas Dörner

**Affiliations:** ^1^Department of Nephrology and Intensive Medical Care, Charité- Universitätsmedizin Berlin, Berlin, Germany; ^2^Department of Rheumatology and Clinical Immunology, Charité- Universitätsmedizin Berlin, Berlin, Germany; ^3^Deutsches Rheumaforschungszentrum, Berlin, Germany; ^4^Grupo de Inmunología Celular e Inmunogenética, Facultad de Medicina, Instituto de Investigaciones Médicas, Universidad de Antioquia UdeA, Medellín, Colombia; ^5^Roche Pharma Research and Early Development, Immunology, Infectious Diseases and Ophthalmology (I2O) Discovery and Translational Area, Roche Innovation Center Basel, Basel, Switzerland; ^6^Roche Pharma Research and Early Development, Pharmaceutical Sciences, Roche Innovation Center Basel, F. Hoffmann-La Roche Ltd, Basel, Switzerland; ^7^Department of Rheumatology and Clinical Immunology, Faculty of Medicine, Medical Center-University of Freiburg, Freiburg im Breisgau, Germany; ^8^Center for Chronic Immunodeficiency (CCI), Faculty of Medicine, Medical Center - University of Freiburg, Freiburg im Breisgau, Germany; ^9^Berlin Institute of Health, Charité Universitätsmedizin Berlin, Berlin, Germany

**Keywords:** B cell, SLE, pSS, lupus, CD11c, atypical B cells, CD21

## Abstract

Circulating CD11c^+^ B cells are a key phenomenon in certain types of autoimmunity but have also been described in the context of regular immune responses (i.e., infections, vaccination). Using mass cytometry to profile 46 different markers on individual immune cells, we systematically initially confirmed the presence of increased CD11c^+^ B cells in the blood of systemic lupus erythematosus (SLE) patients. Notably, significant differences in the expression of CD21, CD27, and CD38 became apparent between CD11c^−^ and CD11c^+^ B cells. We observed direct correlation of the frequency of CD21^−^CD27^−^ B cells and CD21^−^CD38^−^ B cells with CD11c^+^ B cells, which were most pronounced in SLE compared to primary Sjögren's syndrome patients (pSS) and healthy donors (HD). Thus, CD11c^+^ B cells resided mainly within memory subsets and were enriched in CD27^−^IgD^−^, CD21^−^CD27^−^, and CD21^−^CD38^−^ B cell phenotypes. CD11c^+^ B cells from all donor groups (SLE, pSS, and HD) showed enhanced CD69, Ki-67, CD45RO, CD45RA, and CD19 expression, whereas the membrane expression of CXCR5 and CD21 were diminished. Notably, SLE CD11c^+^ B cells showed enhanced expression of the checkpoint molecules CD86, PD1, PDL1, CD137, VISTA, and CTLA-4 compared to HD. The substantial increase of CD11c^+^ B cells with a CD21^−^ phenotype co-expressing distinct activation and checkpoint markers, points to a quantitative increased alternate (extrafollicular) B cell activation route possibly related to abnormal immune regulation as seen under the striking inflammatory conditions of SLE which shows a characteristic PD-1/PD-L1 upregulation.

## Introduction

B cells play a key role in adaptive immunity through their ability to produce antibodies and through several antibody-independent functions such as antigen-presentation and immune regulation. In chronic autoimmune diseases, such as systemic lupus erythematosus (SLE) and primary Sjögren's syndrome (pSS), a variety of B cell disturbances have been described, among them, increased CD11c^+^ B cells (1–3). This population, which is not uniformly defined yet (4), has been proposed to be a source of autoreactivity in autoimmune diseases (1). In mice CD11c expression in B cells is promoted by IFN-γ and IL-21 and antagonized by IL-4 in TLR7 and TLR9 stimulated B cells (5). In healthy donors (HD), it has been reported that CD11c^+^ B cells in blood display a memory phenotype (6). The current conventional definition of human circulating memory B cells is based on CD27 and IgD expression to assign memory B cells to CD27^+^IgD^+^, CD27^+^IgD^−^ and CD27^−^IgD^−^ (“double negative,” DN) subsets. As a further marker CD21 has been suggested to reflect the activation status of memory B cells. CD21 is downregulated in conditions of chronic immune stimulation, e.g., autoimmune diseases like SLE (7) and rheumatoid arthritis (8), in primary immunodeficiency associated with immune dysregulation (9), infections like HIV (10) and malaria (11), among others.

In this study, we applied a comprehensive integrated phenotyping using 46 different markers to study CD11c^+^ B cells in healthy donors, pSS and SLE patients in order to identify disease-related quantitative and qualitative differences of this cellular subset. As a result, we found that CD11c^+^ B cells are in their majority CD21^−^ and belong to the memory compartment. CD11c^+^ B cells display a distinct phenotype with an increased expression of activation and checkpoint inhibitory markers. In autoimmune patients and in particular in SLE, CD11c^+^ B cells were characteristically enriched among CD27^−^IgD^−^, CD27^−^CD21^−^ as well as CD21^−^CD38^−^ compartments, alongside with increased expression of some activation and checkpoint inhibitory markers compared to HD in particular in SLE followed by pSS. These findings point to circulating CD11c^+^ CD21^low^ B cells, which abundance is increased in the blood of SLE and pSS patients, as disease-related peripheral B cells, likely generated in extrafollicular immune reactions and escaping physiological immune regulation.

## Materials and Methods

### Study Participants

Blood was drawn from 18 healthy donors (HD), 27 SLE patients and 22 pSS patients in Lithium Heparin (LiHep)-anticoagulated and EDTA-anticoagulated tubes (Greiner Bio-One). Detailed information on patient demographics as well as clinical and serological parameters are summarized in Supplementary Tables 1–3. The study has been approved by the local ethics committee of Charité Universitätsmedizin Berlin and written consent was obtained from all patients and healthy donors.

### Whole Blood Stainings of Major Subsets and CD169/SIGLEC-1

Absolute numbers of T, B, and NK cells were assessed with BD Multitest 6-color TBNK according to the manufacturer's instructions (BD Biosciences) using a BD FACS Canto II flow cytometer. Lysis of EDTA-anti-coagulated blood and staining for CD169/SIGLEC-1 expression on CD14^+^ monocytes was performed as described (12, 13).

### Isolation and Storage of Mononuclear Cells

Peripheral blood mononuclear cells (PBMCs) were isolated from Li-Hep-anticoagulated blood as described previously (14). Briefly, blood was mixed with PBS (Biochrom) and subjected to density gradient centrifugation with Ficoll Paque Plus (GE Healthcare). Collected mononuclear cells were washed twice with PBS and counted for further experiments. For subsequent stainings, up to 10 × 10^6^ PBMCs were transferred to FBS/10% DMSO, frozen to −80°C in a CoolCell Cell Freezing Container (Biocision) and stored at −80°C until further processing.

### Staining and CyTOF Acquisition

A total of 2 × 10^6^ PBMCs per sample were plated into 96-well v bottom plates and washed with PBS (Fluidigm). Cells were resuspended in 100 μL cisplatin solution (1:2,500 in PBS; Fluidigm) and incubated for 3 min at room temperature. Fifty microliters cell staining buffer (CSB; Fluidigm) was added to quench residual Cisplatin. Cells were washed twice with CSB and fixed with Fix I buffer (1:5 in PBS; Fluidigm) for 10 min at room temperature. Cells were washed with CSB and incubated in 100 μL Barcode/Perm buffer (1:10 in PBS; Fluidigm) supplemented with 2 μL of barcoding reagent (Fluidigm) for 10 min at room temperature. Subsequently, cells were washed with Barcode/Perm buffer and with CSB and pooled in a 1.5 mL low protein binding conical tube (Eppendorf). Twenty microliters of Fc-blocking reagent (130-059-901; Miltenyi Biotec) was added to the cell pellet. For each duplicated, cells were resuspended in 300 μL of one of the staining cocktails in PBS (Supplementary Table 4) and incubated for 30 min at room temperature. Overall, 46 markers could be analyzed in two stainings with a common backbone of 22 markers. Thereafter, cells were washed twice with CSB, resuspended in 500 μL permeabilization buffer (1:10 in water; 00-8333-56; eBioscience) and incubated for 30 min at 4°C. To block unspecific binding of antibodies to intracellular targets, cells were blocked with 1% normal rabbit serum (31883) and 1% normal mouse serum (31880; both Invitrogen) in permeabilization buffer for 15 min at 4°C. Antibodies were added in a total volume of 300 μL in the presence of 1% rabbit serum and 1% mouse serum and incubated for 60 min at 4°C. Cells were washed twice with permeabilization buffer and once with CSB prior to overnight storage in 1 mL intercalator solution (1:6,000 in Perm/Fix buffer; Fluidigm). The next day, cells were washed three times with CSB and twice with water to remove any residual protein. Cell count was determined and cells were diluted with water to a final concentration of 7 × 10^5^ cells/mL. Prior to acquisition, cells were filtered through a 35 μm cell strainer (352235, Corning) and calibration beads (201078, Fluidigm) were added at a 1:10 ratio (v/v). A Helios mass cytometry instrument (Fluidigm) was used for sample acquisition. Data analysis was performed using the Cytobank platform. The data were obtained and analyzed according to the “Guidelines for the use of flow cytometry and cell sorting in immunological studies” (15).

### Data Analysis and Statistics

Flow cytometry data were analyzed using FACSDiva software (Becton Dickinson) and FlowJo (version 10.6.1, TreeStar). For the presentation of t-SNE plots, a concatenated file composed of 22.500 B cell from 15 HD, 15 pSS, and 15 SLE (500 downsampled B cells per donor) with staining panel B was used to ensure robust comparison of data into both the subsets and the cohorts. Statistical analyses and graphical presentations were carried out by using GraphPad Prism Software (GraphPad). For multiple group comparison two-way ANOVA with Šidák's post-test for multiple comparison was used. Spearman correlation coefficient was calculated to detect possible associations between parameters or with disease activity. *P*-values were considered significant when < 0.05. Correlation matrix was calculated using base R and corrplot package (R Foundation for Statistical Computing) using Kendall method. Only correlations with *p* ≤ 0.01 were plotted in the matrix.

## Results

### Higher Frequency of CD11c^+^ B Cells in SLE

**Figure 1:**
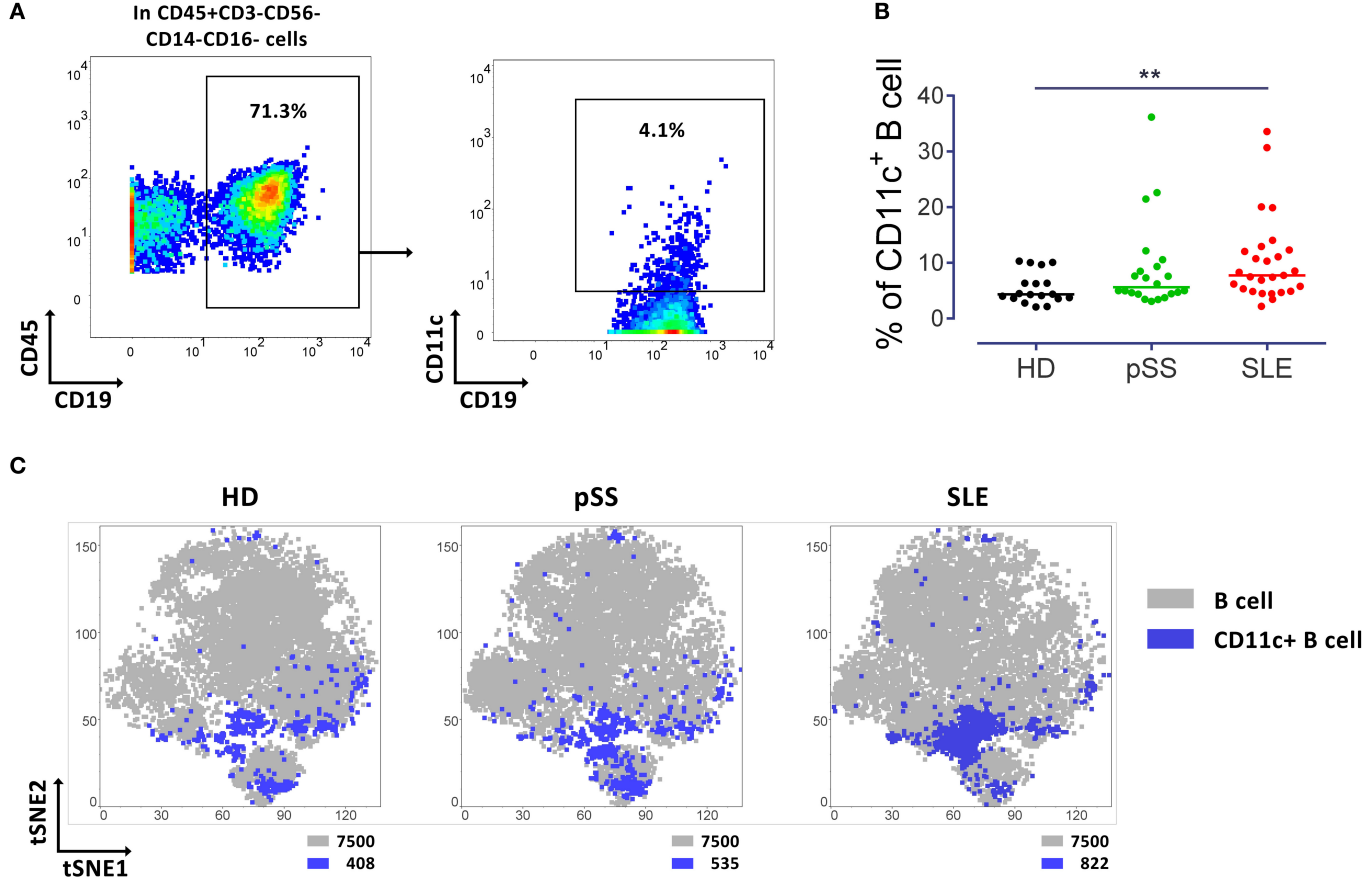
Increased CD11c^+^ B cells in SLE patients. **(A)** Representative pseudocolor plots of B cells (left) and CD11c^+^ B cells (right) from a control (HD). **(B)** Median of frequency of CD11c^+^ B cells from 18 HD, 22 pSS and 27 SLE patients, each point represents a donor. Kruskal-Wallis test with Dunn's post-test. **p < 0.01. **(C)** t-SNE plots of CD11c^+^ B cells (Blue) and general B cell (gray) populations. t-SNE was performed from concatenated file composed from 15 HD, 15 pSS and 15 SLE (7,500 events per group). Number of events of CD11c^+^ and B cells are indicated for each t-SNE plot.

To address the frequency, distribution, and phenotype of peripheral CD11c^+^ B cells, this study comprehensively analyzed these characteristics among peripheral blood cells from 18 healthy donors, 22 pSS patients and 27 SLE patients by mass cytometry, for the expression of overall 46 cell surface proteins. Gating of CD11c^+^ B cells is shown in Figure 1A. The frequency of CD11c^+^ B cells was significantly increased in SLE patients compared to HD (*p* < 0.01; Figure 1B). This observation validated that the population of interest is increased in SLE, consistent with conventional flow cytometry data (1). Interestingly, two-dimension t-SNE plots clustering all peripheral B cells according to expression patterns of the parameters analyzed (Figure 1C), showed a similar qualitative distribution of CD11c^+^ B cells among the two patient groups and healthy controls. However, CD11c^+^ B cells were slightly increased in pSS (535 events) and showed a significant enrichment in SLE patients (822 events), as compared to HD (408 events) (Figure 1C) when normalized to an acquisition of 7,500 B cells concatenated from 15 randomly selected individuals of each group.

### Circulating CD11c^+^ B Cells Lacking CD21 and CD27 Expression Are Enhanced in SLE and pSS Patients

**Figure 2:**
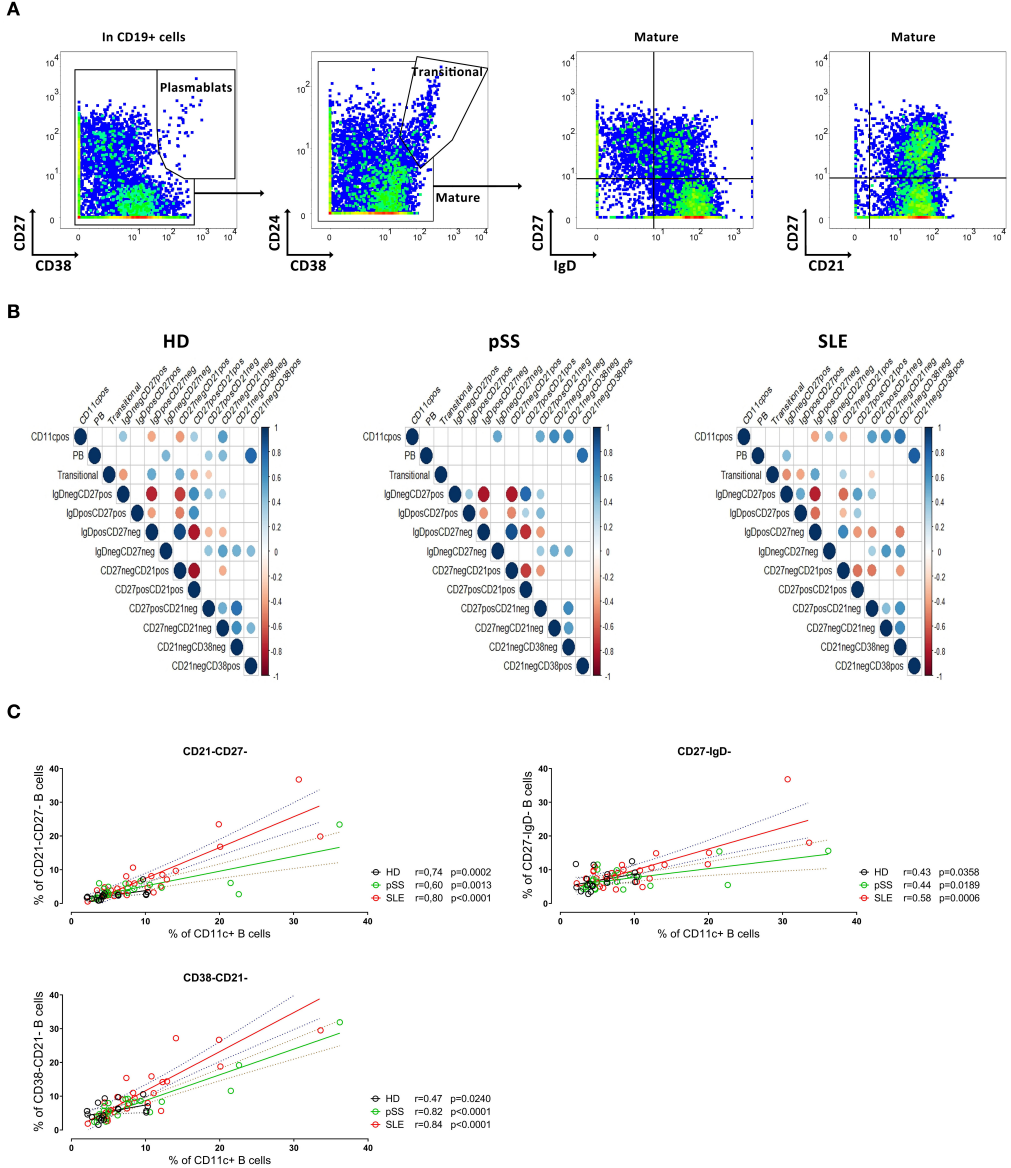
Subset characterization of CD11c^+^ B cells. **(A)** Representative pseudocolor plots of gating of CD19^+^ B cells into plasmablasts (left), transitional and mature B cells (middle left), and representative pseudocolor plots of IgD/CD27 based classification (middle right) and CD21/CD27 based classification of mature B cells (left) from a HD. **(B)** Kendall correlation matrix showing the correlation of frequency of each B cell subset (as shown in **A**) together with CD11c^+^ B cells from 18 HD (left), 22 pSS (center), and 27 SLE (right). Correlations are represented by red (negative) or blue (positive) circles, referring the size and intensity of color to the strength of correlation. Only correlations with *p* ≤ 0.01 are indicated. **(C)** Spearman rank correlation between CD11c^+^ with CD21^−^CD27^−^ (upper left), CD21^−^CD38^−^ (lower panel left) and CD27^−^IgD^−^ (upper panel right) populations from 18 HD, 22 pSS, and 27 SLE, respectively. Each point represents a donor.

Initial analyses addressed the frequency of CD11c^+^ B cells among certain B cell subsets classified into plasmablast (CD27^+^CD38^+^), transitional (CD24^+^CD38^+^) and mature populations (Figure 2A). Mature B cells were divided according to CD27 and IgD into CD27^−^IgD^+^, CD27^+^IgD^+^, CD27^+^IgD^−^ and double negative (DN) CD27^−^IgD^−^ subsets (Figure 2A); or related to their CD21 and CD27 expression into CD21^+^CD27^−^, CD21^+^CD27^−^, CD21^−^CD27^+^, and CD21^−^CD27^−^ B cells (Figure 2A).

To evaluate the relationship between CD11c^+^ B cells, plasmablasts, transitional and mature B cells (the latter classified according to IgD/CD27 and CD21/CD27 expression), a correlation matrix was obtained from the three cohorts (Figure 2B). Of interest, a significant positive correlation between the frequencies of CD21^−^CD27^−^ and those of CD11c^+^ B cells was observed for all patient groups as well as HD. Interestingly, there was also a correlation between the frequencies CD11c^+^ B cells with CD27^−^IgD^−^ as well as CD27^−^CD38^−^ B cells for pSS and SLE patients, respectively, but not for HD (Figure 2B).

Further analyses revealed a correlation between the frequencies of CD11c^+^ and CD21^−^CD27^−^ B cells both from HD (*r* = 0.74, *p* = 0.0002), pSS (*r* = 0.60, *p* = 0.0013) and SLE patients (*r* = 0.80, *p* < 0.0001) (Figure 2C). In addition, frequencies of CD21^−^CD38^−^ B cells closely correlated with those of CD11c^+^ B cells from all three cohorts (Figure 2C, see next section). No correlation was found for the frequencies of CD11c^+^ B cells and the age of the individual donors, the expression of Siglec-1 (CD169) on monocytes (as marker of the type I interferon signature) or the disease activity scores SLEDAI for SLE and ESSDAI for pSS, respectively (data not shown). Thus, the data indicate that CD11c^+^ B cells are more intimately related but not restricted to atypical memory B cells lacking CD21 and/or CD38 expression prompting additional studies.

### CD11c^+^ B Cells Are Characteristically Enriched Within the Atypical B Cells Lacking Expression of CD21 and CD38

**Figure 3:**
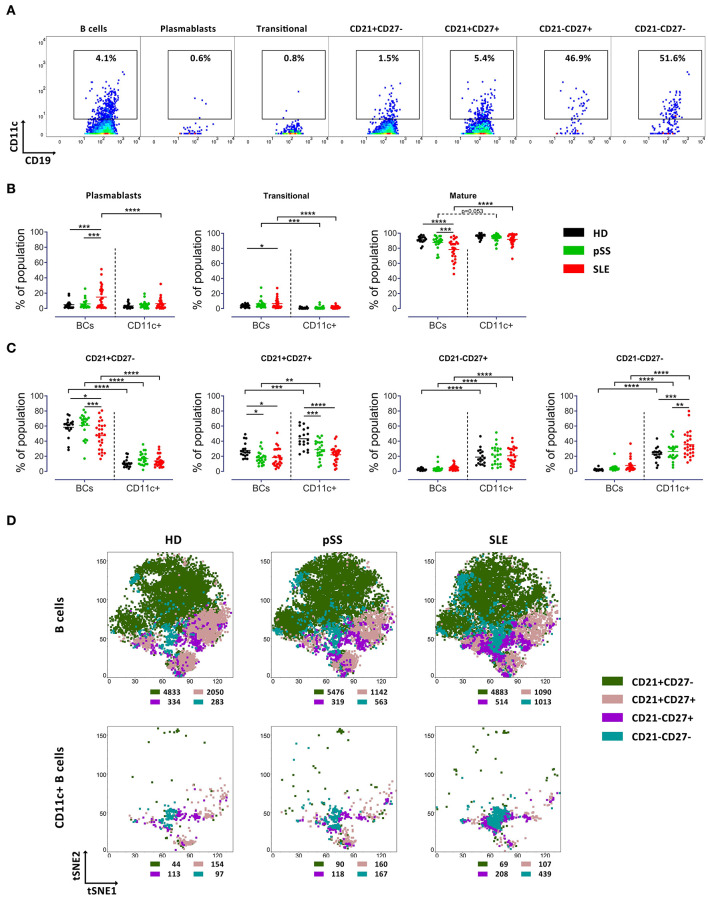
Increased frequency of CD11c^+^ B cells carrying CD21^−^CD27^−^ phenotype in SLE patients. **(A)** Representative pseudocolor plots of CD11c expression by B cell subsets (in CD19^+^ B cells, plasmablasts, transitional B cells, CD21^+^CD27^−^, CD21^+^CD27^+^, CD21^−^CD27^+^, and CD21^−^CD27^−^ populations from left to right) from a HD. **(B)** Median of frequency of plasmablasts, transitional B cells and mature B cells in overall B cells and CD11c^+^ B cells from 18 HD, 22 pSS and 27 SLE patients, each point represents a donor. Two way ANOVA with Šidák's post-test. *p < 0.05, **p < 0.01, ***p < 0.001, ****p < 0.0001. **(C)** Median frequency of CD21/CD27 based phenotypes in mature B cells and CD11c^+^ mature B cells from 18 HD, 22 pSS and 27 SLE patients, each point represents a donor. Two-way ANOVA with Šidák's post-test. *p < 0.05, **p < 0.01, ***p < 0.001, ****p < 0.0001. **(D)** t-SNE plots of CD21^+^CD27^−^, CD21^+^CD27^+^, CD21^−^CD27^+^, and CD21^−^CD27^−^ populations in general B cells (upper panel) and CD11c^+^ B cells (lower panel) from HD (left), pSS (middle) and SLE (right) patients. t-SNE was performed from concatenated files composed from 15 HD, 15 pSS and 15 SLE (7,500 events per group), respectively. The number of events of each subset in B cells and CD11c^+^ cells are indicated for each t-SNE plot.

Plasmablasts (CD38^+^CD27^+^), transitional (CD38^high^CD24^high^) and CD21^+^CD27^−^ B cells showed lower frequency of CD11c^+^ B cells than the overall B cell population. The remaining three populations exhibited higher frequencies of CD11c^+^ cells (Figure 3A) that were even higher than among CD27^−^IgD^−^ B cells (Supplementary Figure 1A). Thus, the majority of CD11c^+^ B cells carried a mature phenotype (>80%). Frequencies of plasmablasts and transitional subsets among CD11c^+^ and global B cell population were similar, with no differences between the study groups (Figure 3B).

Subsequently, mature B cells and CD11c^+^ mature B cells were classified according to their expression of CD27 and IgD (Figure 2A). In general, frequencies of CD11c^+^ B cells were enhanced among all memory B cell subsets across all cohorts, and low among CD27^−^IgD^+^ naïve B cells (Supplementary Figure 1A). Interestingly, differences between SLE and pSS patients and HD were found for the three memory subsets (CD27^+^IgD^+^; CD27^+^IgD^−^ and CD27^−^IgD^−^). In particular, an increased frequency of CD11c^+^ B cells carrying a DN (CD27^−^IgD^−^) phenotype in SLE compared to pSS (*p* < 0.01) and HD (*p* < 0.0001) was observed, while lower frequencies of CD11c^+^ B cells with CD27^+^IgD^+^ (pSS and SLE vs. HD) and CD27^+^IgD^−^ (SLE vs. pSS and HD) were identified (Supplementary Figure 1C). Comparison of t-SNE distribution supported the enrichment of CD11c^+^ B cells among memory subsets, but especially within CD27^−^IgD^−^ B cells in SLE patients (Supplementary Figure 1C).

CD21 has been described to be related to CD11c expression linked to B cell activation, autoimmunity and as part of age associated B cells phenotype (16–19). A classification of B cells according to their CD21 and CD38 expression in CD21^+^, CD21^−^CD38^−^ and CD21^−^CD38^+^ further revealed the characteristics of CD11c^+^ B cells (Supplementary Figure 2A). While CD11c^+^ B cells were substantially lower among CD21^+^ B cells, with marked differences between the cohorts (Supplementary Figure 2B), there was a remarkable enrichment of CD11c^+^ B cells among CD21^−^CD38^−^ B cells. Frequencies of CD21^−^CD38^−^ B cells showed a weak correlation with those of CD11c^+^ B cells in HD (*r* = 0.47, *p* = 0.024), but strong correlations in pSS (*r* = 0.82, *p* = 0.0001) and SLE (*r* = 0.84, *p* < 0.0001), respectively (Figure 2C). This observation suggests a potential role for the phenotype of CD21^−^CD38^−^ B cells during pronounced immune activation as seen in SLE and to a lesser extent for pSS in contrast to controls.

The frequencies of CD11c^+^ B cells expressing either CD21 and those which were negative for CD21 and CD38 were similar in HD (Supplementary Figure 2B). CD11c^+^ B cells appeared to be enriched in particular among CD21^−^CD27^+^ and CD21^−^CD27^−^ B cells, compared to the overall B cell population (Figure 3A). Compared to the overall B cell population, CD11c^+^ B cells resided largely among CD21^−^ subsets (CD21^−^CD27^+^and CD21^−^CD27^−^) in all three cohorts. Of particular note, frequencies of CD11c^+^ B cells carrying the CD21^−^CD27^−^ phenotype were increased in SLE compared to HD (*p* < 0.001) and pSS (*p* < 0.01) (Figure 3C). Comparison of t-SNE distribution of general mature B cells and the CD11c^+^ mature B cell population among CD21^+^CD27^−^, CD21^+^CD27^+^, CD21^−^CD27^+^, and CD21^−^CD27^−^ populations of the cohorts is shown in Figure 3D. Here, the characteristic enrichment of CD11c^+^ B cells among CD21^−^CD27^−^ B cells and within CD21^−^CD38^−^ B cells (Supplementary Figure 2C) as characteristics of these cells in SLE became clearly evident (Figure 3D).

### CD11c^+^ B Cells Express Checkpoint Molecules Carry - Increased PD-1 and PD-L1 Mark CD11c+ B Cells in SLE

**Figure 4:**
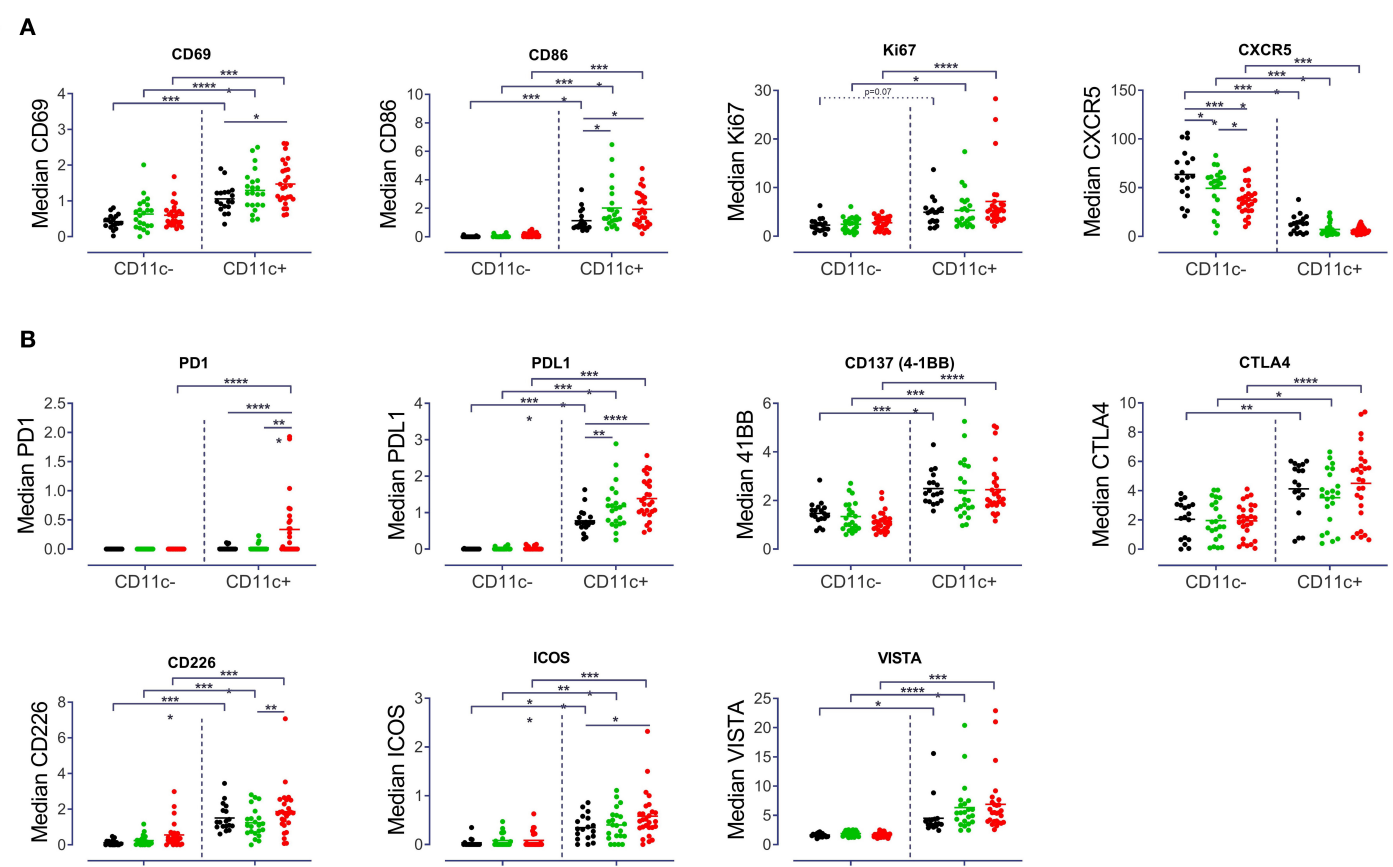
Distinct expression pattern by CD11c^+^ B cells. Median signal intensity of **(A)** Activation and proliferation markers (CD69, CD86, Ki67, and CXCR5), **(B)** Checkpoint molecule expression (PD1, PDL1, CD137, CTLA4, CDD226, ICOS, and VISTA) among CD11c^−^ and CD11c^+^ from 18 HD, 22 pSS and 27 SLE patients, each point represents a donor. Two-way ANOVA with Šidák's post-test. *p < 0.05, **p < 0.01, ***p < 0.001, ****p < 0.0001.

A particular goal of this study was to identify the checkpoint molecule expression profile by CD11c^+^ B cells in autoimmunity. As a result, CD11c^+^ B cells showed a higher activation status as compared to CD11c^−^ B cells, with increased expression of CD69 (*p* < 0.001) and CD86 (*p* < 0.001) across all three groups of donors (Figure 4A). CD69 expression of SLE B cells (*p* < 0.05) and CD86 expression of B cells from pSS (*p* < 0.05) and SLE patients (*p* < 0.05) were significantly increased for CD11c^+^ B cells compared to HD (Figure 4A). CD11c^+^ B cells from pSS (*p* < 0.05) and SLE patients (*p* < 0.0001) also showed a higher Ki67 expression, when compared to HD (Figure 4A). Moreover, CD11c^+^ B cells showed diminished expression of CXCR5 (*p* < 0.0001) in all groups compared to CD11c^−^ B cells, suggesting their routing outside of germinal centers. Although a remarkably lower CXCR5 expression has been observed in CD11c^−^ B cells from SLE patients compared to HD (*p* < 0.0001) and pSS (*p* < 0.05) patients, respectively, this alteration was not observed among CD11c^+^ B cells (Figure 4A).

A characteristically higher expression of CD45RO (*p* < 0.001) and CD19 (*p* < 0.01) together with a lower expression of CD38 (*p* < 0.01) by CD11c^+^ B cells from all three groups was detected (Supplementary Figure 3). Furthermore, a higher expression of CD45RA (*p* < 0.01) among CD11c^+^ B cells from HD and pSS but not SLE was found (Supplementary Figure 3). In addition, CD45RA expression was lower on CD11c^+^ B cells from SLE compared to HD (Supplementary Figure 3). No differences in IgA, IgG, and IgM expression between CD11c^+^ and CD11c^−^ or between study groups were found (Supplementary Figure 3).

Analysis of the expression of certain checkpoint inhibitors revealed that CD11c^+^ B cells had a significantly increased expression of PDL1 (*p* < 0.0001), CD137 (4-1BB, *p* < 0.001), VISTA (*p* < 0.05) and CTLA4 (*p* < 0.05), as compared to CD11c^−^ B cells (Figure 4B). No striking differences were found for BTLA and PSGL-1 between CD11c^+^ and CD11c^−^ B cells and between groups of patients (Supplementary Figure 3). One notable difference in terms of checkpoint inhibitor expression was the increased PD1 expression on CD11c^+^ B cells in SLE compared to CD11c^−^ (*p* < 0.0001) and compared to CD11c^+^ B cells from HD (*p* < 0.0001) and pSS (*p* < 0.001) patients (Figure 4B). An increased PDL1 expression was observed among CD11c^+^ B cells for pSS (*p* < 0.01) and SLE (*p* < 0.0001) compared to controls (Figure 4B). Overall, enhanced expression of a distinct pattern of checkpoint molecules (PDL1, CD137, CTLA4, CD226, ICOS, and VISTA) was a characteristic of CD11c^+^ B cells, across all groups studied (Figure 4B). There were no differences in data of patients classified according to disease activity score, medication and organ involvement (Supplementary Figure 4 and data not shown).

In summary, CD11c^+^ B cells are enhanced among memory B cell subsets with reduced CD21 and CD38 expression, and a characteristic profile of CD69, CD86, PDL1, CD137, and CTLA4 expression. Substantially increased PD1 together with elevated PD-L1expression on CD11c^+^ B cells was found as a characteristic and unique finding of SLE CD11c^+^ B cells.

## Discussion

Although CD11c^+^ B cells have been suggested as a key player in autoimmunity, a comprehensive analysis of them including detailed studies of their checkpoint molecule expression between different autoimmune conditions had not been undertaken so far. In this study we have used mass cytometry-based immunophenotyping to that end. We observed an increased frequency of CD11c^+^, mostly CD21^−^ B cells among all circulating B cells of SLE patients. CD11c^+^ B cells of SLE and pSS patients showed a number of phenotypic differences. In the literature the terms of CD11c^+^ and CD11c^high^ are often not sharply separated while it is important to note that CD11c^high^ B cells are only a fraction of total CD11c^+^ B cells. Both populations have been described to be increased in SLE patients (1), but also under physiological conditions of immune challenges, such as infections and in response to vaccinations (20).

CD11c^+^ circulating B cells are enriched among CD21^+^CD27^+^, CD21^−^CD27^+^ and especially CD21^−^CD27^−^ B cells, as compared to all B cells. A striking correlation of CD11c^+^ and CD21^−^CD27^−^ expression was observed for B cells of all the three study groups, but was especially enriched in SLE. A significant correlation was found for CD11c^+^ and CD21^−^CD38^−^ B cells in SLE and pSS patients, while the correlation was less among controls. Circulating CD21^−^ or CD21^low^ B cells have been described to be increased in frequency in various autoimmune diseases like SLE (21), rheumatoid arthritis (17) and pSS (22), as well in other conditions, like malaria (11), COVID19 (23), CVID (19), chronic graft-versus-host disease (24) hepatitis C (25) and in HIV infection (15), reflecting chronic activation of the immune system. A common denominator among these conditions is the continued egress of activated B cells into the blood. In this context, reduced CXCR5 expression can be considered as another marker of recent activation and/or B cell differentiation outside the conventional GC differentiation route (26). It has been reported for mice, that CD11c^+^ cells are located at the border of T cell to B cell zones in the spleen (27). The reduced expression of CXCR5 by CD11c^+^ B cells described here, argues in favor of the extrafollicular generation of CD11c^+^ B cells in autoimmunity, especially in active disease, and the enrichment of autoreactive clones due to the lack of proper selection outside germinal centers. It is important to note that CD11c^+^ B cells were enriched among, but not limited to CD21^−^B cells. The data are consistent with a quantitative increase of atypical memory B cells carrying the phenotype of CD11c lacking the expression of CD21 and CD38 which are possibly induced outside the GCs with impaired selection under the condition of SLE followed by pSS and HD.

CD21 expression of CD11c^+^ B cells was markedly diminished in all studied donor groups, but most significantly among SLE patients. CD11c^+^ B cells were mainly enriched, but not limited, to the memory B cell phenotypes CD27^+^IgD^+^, CD27^+^IgD^−^, and CD27^−^IgD^−^(DN), as previously reported (6), indicating that CD11c^+^ expression is induced by B cell activation. An increased frequency of DN B cells is a hallmark of SLE (7, 28, 29) and has also been described in rheumatoid arthritis (30) and multiple sclerosis (31). Increased frequency of CD11c^+^ among the CD27^−^IgD^−^ phenotype in SLE patients could be explained by a combination of enrichment of CD11c^+^ cells (observed in both HD and patients) as well as the increased DN phenotype in total B cells in SLE patients. It is worth noting, that DN CD11c^+^ B cells are more frequent among memory B cell subsets in SLE, as compared to pSS and HD. The differences in the expression of markers in CD11c^+^ compared to CD11c^−^ B cells, showed most pronounced changes in SLE B cells and to a lesser extent among pSS patients. The data suggest that the mechanisms involved in the generation of CD11c^+^ B cells are common across all cohorts but their amplification in SLE and pSS appear to gain pathogenic importance.

A key objective of the study was the characterization of checkpoint molecule expression in connection to other markers by CD11c^+^ B cells to gain additional insights. Here, an increased expression of activation (CD69, CD86), proliferation (Ki67), pro-survival (CD137) and co-inhibitory (PD1, PDL1) markers was found. In addition, enhanced expression of CTLA4, CD226, ICOS, and VISTA was found as a characteristic of CD11c^+^ B cells across all groups studied. These markers together with reduced CD21 are known to be induced upon B cell stimulation (32–35), suggesting that CD11c^+^ are antigen-experienced and recently activated cells. In addition to increased expression of CD69 and CD86, SLE CD11c^+^ B cells carried a characteristically increased PD1 and PD-L1 expression, suggesting that they result from a dysregulation in SLE. After B cell stimulation an increased expression of CD86, PD1 and PDL1 in B cells from HD and SLE patients has been reported (33) supporting the idea that CD11c^+^ B cells had previously been stimulated *in vivo* and their generation is promoted in autoimmune conditions. CD11c^+^ B cells of pSS patients also showed increased CD86 and PDL1 and reduced CD27 expression, displaying a similar B cell disturbance but less severe than SLE patients. Previous studies had shown increased CD86 expression by all subsets of peripheral B cells (36), while others described an increase in CD27^+^IgD^−^ and DN, but not in naïve or CD27^+^IgD^+^ B cell from pSS (37). Increased PDL1 had been detected in salivary glands of pSS patients (38) while data on PDL1 expression on pSS B cells are missing. It remains to be further delineated whether these findings result from their more extensive generation in SLE or really reflect a unique abnormality in lupus.

In autoimmune diseases and infections, excessive activation of B cells leads to an accumulation of exhausted/post-activated peripheral B cells, characterized by hyporesponsiveness to activating stimuli and upregulated expression of regulatory markers, like PD1 and PDL1 (25, 39, 40). CD21 and CD11c both have been described to play a role on chronic cognate activation of B cells, and they are linked in several ways: Transcripts of ITGAX, encoding CD11c, are upregulated in CD21^−^ B cells, which qualify as memory B cells due to their class switched BCRs (16), or as anergic B cells due to their lower activation threshold and calcium mobilization upon B cell stimulation and their autoreactive BCR repertoire (17). Age-associated B cells (ABCs) initially had been described in mice and described as antigen-experienced and refractory to BCR stimuli (18). CD11c^+^CD21^−^ B cells have been described as the human equivalent of ABCs, their frequencies increased in rheumatoid arthritis, and correlating with patients' age (41). Noteworthy, in our cohort frequencies of CD11c^+^ cells and age did not correlate, which is in line with a previous publication showing no correlation between CD11c^high^ B cell and age of SLE patients (1). Whether ABCs do really represent a distinct B cell lineage or whether they rather represent a differentiation status of antigen-experienced B cells in chronic immune reactions is debatable (4).

A limitation of the study aiming on the characterization of checkpoint molecule expression by CD11c^+^ B cells is that CD21 was only included in one of two panels used, which limited the characterization of expanded CD11c^+^ B cells. Other limitation is the detection of low signals in some markers which should ideally be verified by other methods. Here we describe that peripheral CD11c^+^ B cells are heterogeneous in phenotype, but many of them show reduced CD21 and CD38 expression, including upregulated expression of a distinct pattern of checkpoint molecules (PD-1, PDL1, CD86, CD137, CTLA4, CD226, ICOS, and VISTA). Increased PD-1 and PD-L1 expression was characteristic of SLE CD11c^+^ B cells. This suggests that CD11c^+^ B cell induction depends quantitively on the extent of immune activation but can result in qualitative differences of abnormal and likely dysregulated immune activation as exemplified by CD11c^+^ B cells carrying increased PD-1 and PD-L1 in SLE.

## Data Availability Statement

The raw data supporting the conclusions of this article will be made available by the authors, without undue reservation.

## Ethics Statement

The studies involving human participants were reviewed and approved by EA1/302/16. The patients/participants provided their written informed consent to participate in this study.

## Author Contributions

The concept of the study was developed by HR-A, ES, and TD. Data were obtained by AW, LS, ML, FS, APF, TK-T, and MS. Data were analyzed by HR-A, ES, TK-T, and MS. The theoretical framework was developed by TD, ES, AL, KW, BK, and AR. All authors developed, read, and approved the manuscript.

## Conflict of Interest

APF, MS, KH, SF, and TK-T are employed by F. Hoffmann-La Roche AG. The remaining authors declare that the research was conducted in the absence of any commercial or financial relationships that could be construed as a potential conflict of interest.
