# Supplementary material for: Deep Phenotyping of CD11c+ B Cells in Systemic Autoimmunity and Controls
Source: Front Immunol. 2021 Mar 12;12:635615. doi: 10.3389/fimmu.2021.635615 (PMC7994903; doi:10.3389/fimmu.2021.635615)
Supplement: Supplementary file 1 [file Data_Sheet_1.docx]

Supplementary Material

1. **Supplementary figures.**
   1. **Supplementary Figure 1. Increased frequency of CD11c^+^ B cells carrying CD27^-^IgD^-^ (DN) phenotype in autoimmune patients.**

**A.** Representative pseudocolor plots of CD11c expression by B cell subsets (in total CD19^+^ B cells, plasmablasts, transitional B cells, CD27^-^IgD^+^, CD27^+^IgD^+^, CD27^+^IgD^-^ and CD27^-^IgD^-^ populations from left to right) from a HD. **B.** Median of frequency of IgD/CD27 based phenotypes in mature B cells and CD11c^+^ mature B cells from 18 HD, 22 pSS and 27 SLE patients, each point represents one donor. Two-way ANOVA with Šidák´s post-test. *p<0.05, **p<0.01, ***p<0.001, ****p<0.0001. **C.** t-SNE plots of CD27^-^IgD^+^, CD27^+^IgD^+^, CD27^+^IgD^-^ and CD27^-^IgD^-^ populations into general B cells (top) and CD11c^+^ B cells (under) from HD (left), pSS (center) and SLE (right) patients. t-SNE was performed from concatenated file composed from 15 HD, 15 pSS and 15 SLE (7500 events per group), respectively. The number of events of each subset in B cells and CD11c^+^ cells, respectively are indicated for each t-SNE plot.

- 1. **Supplementary Figure 2. Increased frequency of CD11c^+^ B cells carrying CD21^-^CD38^-^ phenotype in autoimmune patients.**

**A.** Representative pseudocolor plots of B cells and CD11c^+^ B cells according to their CD21 and CD38 expression. **B.** Median of frequency of CD21/CD38 based phenotypes among overall B cells and CD11c^+^ B cells from 18 HD, 22 pSS and 27 SLE patients, each point represents an individual donor. Two-way ANOVA with Šidák´s post-test. *p<0.05, **p<0.01, ***p<0.001, ****p<0.0001. **C.** t-SNE plots of CD21^+^, CD38^+^CD21^-^ and CD38^-^CD21^-^ populations among overall B cells (upper panel) and CD11c^+^ B cells (lower panel) from HD (left), pSS (middle) and SLE (right) patients. t-SNE was performed from concatenated file composed from 15 HD, 15 pSS and 15 SLE (7500 events per group), respectively. The number of events of each subset among overall B cells and CD11c^+^ cells are indicated for each t-SNE plot.

- 1. **Supplementary Figure 3.**

**Phenotypic characterization of CD11c^+^ B cells related to a panel of additional markers.** Median of Median Fluorescence Intensity (MFI) of the indicated markers from 18 HD, 22 pSS and 27 SLE patients, respectively; each point represents an individual donor. Two-way ANOVA with Šidák´s post-test. *p<0.05, **p<0.01, ***p<0.001, ****p<0.0001.

- 1. **Supplementary Figure 4. Increased frequency of CD11c^+^ B cells carrying CD21^-^CD38^-^ phenotype in autoimmune patients.**

**A.** Median frequency of CD11c^+^ B cells from 18 low activity disease (SLEDAI ≤4) and 9 active disease (SLEDAI >4) SLE patients. **B.** Median Fluorescence Intensity (MFI) of the indicated markers from 18 low activity disease and 9 active disease SLE patients in CD11c^+^ B cells. **C.** Median frequency of CD11c^+^ B cells from 5 SLE patients with arthritis and 7 SLE patients with other organ involvement. **D.** MFI of the indicated markers from 5 SLE patients with arthritis and 7 SLE patients with other organ involvement in CD11c^+^ B cells. **E.** Median frequency of CD11c^+^ B cells from 10 SLE patients without Prednisone treatment and 17 with Prednisone treatment. **F.** MFI of the indicated markers from 10 SLE patients without Prednisone treatment and 17 with Prednisone treatment in CD11c^+^ B cells. Two-way ANOVA with Šidák´s post-test.

# Supplementary Tables

## Supplementary Table 1. Demographic data of the healthy donors and patient groups studied

|  | **HD** | **pSS** | **SLE** |
| --- | --- | --- | --- |
| **N** | 18 | 22 | 27 |
| **Age ^a^** | 30 (24-60) | 52 (34-78) | 38 (24-65) |
| **Men / Women** | 1 / 17 | 2 / 20 | 2 / 25 |
| **Siglec-1 ^a^** | 362 ± 43 | 600 (0 – 3964) | 1724 (130 – 4349) |
| **Disease activity ^a^** | n.a. | 2.5 (0 – 16) ^b^ | 2 (0 – 12) ^c^ |

^a^ Median (Range)

^b^ ESSDAI

^c^ SLEDAI

n.a. not applicable

## Supplementary Table 2. Clinical data of the pSS cohort

| **ID** | **ESSDAI** | **cESSDAI** | **Age** | **Sex** | **Laboratory values** | | | | **Siglec-1 MFI  (of CD14+)** | **Organ Involvement** | **Medication** | | |
| --- | --- | --- | --- | --- | --- | --- | --- | --- | --- | --- | --- | --- | --- |
|  |  |  |  |  | **ANA [Titer]** | **α-dsDNA [U/ml]** | **α-Ro** | **α-La** |  |  | **Prednisolone (mg/d)** | **HCQ (mg/d)** | **Other** |
| **pSS01** | 7 | 7 | 48 | f |  |  |  |  | 670 | constitutional, articular |  |  |  |
| **pSS02** | 6 | 6 | 63 | f | 1:640 | pos | pos | neg | 413 | glandular swelling, articular, hematologic |  |  |  |
| **pSS03** | 7 | 6 | 59 | f |  |  |  |  | 325 | glandular swelling, articular | 5 | 400 |  |
| **pSS04** | 5 | 5 | 60 | f | 1:320 | pos | pos | pos | 766 | constitutional, glandular |  |  |  |
| **pSS05** | 3 | 2 | 36 | f | 1:5120 | pos | pos | pos | 346 | arthralgia | 5 | 200 |  |
| **pSS06** | 2 | 2 | 45 | f | 1:1260 | pos | pos | pos | 1456 | hematologic | 1 | 400 |  |
| **pSS07** | 2 | 2 | 34 | f | 1:160 | pos | pos | neg | 694 | arthralgia |  | 200 |  |
| **pSS08** | 4 | 4 | 46 | f | 1:2560 | pos | pos | pos | 1091 | hematologic, arthralgia | 5 | 400 |  |
| **pSS09** | 0 | 0 | 39 | f | 1:320 | pos | pos | neg | 3471 |  |  |  |  |
| **pSS10** | 1 | 0 | 38 | f | 1:160 | pos | pos | neg | 294 |  |  | 200 |  |
| **pSS11** | 0 | 0 | 55 | f | 1:640 | pos | pos | neg | 0 |  |  | 200 |  |
| **pSS12** | 0 | 0 | 69 | f | 1:10240 | neg | neg | neg | 241 |  |  | 200 |  |
| **pSS13** | 0 | 0 | 59 | f | 1:160 | pos | pos | pos | 295 |  | 5 |  |  |
| **pSS14** | 0 | 0 | 78 | f | 1:2018 | neg | neg | neg | 264 |  |  |  |  |
| **pSS15** | 0 | 0 | 49 | f | 1:640 | pos | pos | pos | 485 |  |  | 300 |  |
| **pSS16** | 1 |  | 34 | f | 1:10240 | pos | pos | pos | 981 |  |  | 200 |  |
| **pSS17** | 1 | 0 | 42 | f | 1:10240 | pos | pos | pos | 721 |  |  | 300 |  |
| **pSS18** | 6 | 4 | 74 | m | 1:2560 | pos | pos | neg | 530 | glandular | 4 |  |  |
| **pSS19** | 14 | 12 | 36 | f | 1:5120 | pos | pos | pos | 2120 | renal, glandular |  |  |  |
| **pSS20** | 16 | 15 | 65 | f | 1:320 | pos | pos | neg | 3964 | severe pulmonary involvement | 5 |  | iv. cyclophosphamide cycles prior to the analysis |
| **pSS21** | 6 | 5 | 69 | m | 1:1280 | pos | pos | neg | 1703 | hematologic | 5 | 200 |  |
| **pSS22** | 6 | 6 | 69 | f | 1:5120 | pos | pos | pos | 275 | glandular, arthralgia |  | 300 |  |

## Supplementary Table 3. Clinical data of the SLE cohort

| **ID** | **Age** | **Sex** | **SLEDAI** | **cSLEDAI** | **Laboratory values** | | | | | | | **Siglec-1 MFI (CD14+ cells)** | **Organ Involvement** | **Medication** | | | | | |
| --- | --- | --- | --- | --- | --- | --- | --- | --- | --- | --- | --- | --- | --- | --- | --- | --- | --- | --- | --- |
|  |  |  |  |  | **ANA [Titer]** | **α-dsDNA [U/ml]** | **α-Sm** | **α-Ro** | **α-La** | **α-U1RNP** | **α-RNP-70** |  |  | **Prednisolone (mg/d)** | **HCQ (mg/d)** | **MMF (mg/d)** | **Azathioprine (mg/d)** | **MTX (mg/week)** | **Other** |
| **SLE01** | 24 | f | 10 | 6 | 1:640 | 75.0 | neg |  |  |  |  | 1220 | arthritis, rash | 5 | 400 |  | 50 | 10 | Belimumab |
| **SLE02** | 51 | f | 6 | 4 | 1:1280 | 44.8 | neg | neg | neg |  |  | 394 | arthritis | 5 | 200 |  |  |  |  |
| **SLE03** | 65 | f | 8 | 8 | 1:640 | 5.0 | neg | neg | neg | neg | neg | 469 | arthritis, rash, ulcers |  |  |  |  |  |  |
| **SLE04** | 50 | f | 12 | 8 | 1:5120 | 37.0 | neg |  |  |  |  | 1203 | arthritis, rash, alopecia |  |  |  |  |  |  |
| **SLE05** | 27 | f | 2 | 2 | 1:160 | 9,6 | neg | neg | neg | neg | neg | 517 | alopecia | 20 |  |  |  |  | Ustekinumab |
| **SLE06** | 63 | f | 2 | 0 | 1:2560 | 71.0 |  |  |  |  |  | 2228 |  |  |  | 500 |  |  |  |
| **SLE07** | 27 | f | 5 | 1 | 1:5120 | 152,4 |  | pos |  | pos | pos | 3984 | leucopenia |  | 400 | 2000 |  |  |  |
| **SLE08** | 41 | f | 2 | 0 | 1:2560 | 84.8 | neg | pos | pos | neg | neg | 1406 |  | 5 | 200 | 1000 |  |  |  |
| **SLE09** | 50 | f | 4 | 0 | 1:1280 | 37,9 | neg | neg | neg | neg | neg | 2019 |  | 7 | 300 |  |  |  | Baricitinib |
| **SLE10** | 39 | f | 4 | 2 | 1:5120 | 27.0 | neg | pos | pos | neg | neg | 3421 | rash | 5 | 400 |  |  | 20 |  |
| **SLE11** | 36 | m | 2 | 0 | 1:640 | 16,7 | neg | neg | neg | pos | neg | 1724 |  | 2.5 | 200 |  |  | 20 |  |
| **SLE12** | 65 | f | 2 | 0 | 1:160 | 32,7 | neg | neg | neg | neg | neg | 130 |  | 4 |  |  | 75 |  | Belimumab |
| **SLE13** | 24 | f | 2 | 0 | 1:1280 | 22,3 | neg | pos | neg | neg | neg | 3343 |  |  |  |  |  |  |  |
| **SLE14** | 35 | m | 2 | 0 | 1:2560 | 13,1 | neg | pos | pos | neg | neg | 3056 |  | 2 |  |  | 75 |  |  |
| **SLE15** | 36 | f | 0 | 0 | 1:1280 | 5,2 | neg | pos | pos | neg | neg | 583 |  | 5 | 200 |  | 50 |  |  |
| **SLE16** | 34 | f | 2 | 0 | 1:1280 | 5,2 | neg | pos | neg | neg | neg | 1878 |  |  |  |  |  |  |  |
| **SLE17** | 39 | f | 2 | 0 | 1:1280 |  | neg | pos | pos | neg | neg | 3440 | antiphospholipid syndrome | 5 | 200 |  |  |  |  |
| **SLE18** | 38 | f | 2 | 0 | 1:160 | 20,5 | neg | pos | neg | neg | neg | 559 |  | 2.5 |  |  |  |  |  |
| **SLE19** | 54 | f | 2 | 0 | 1:2560 | 24,5 | neg | neg | neg | neg | neg | 229 |  | 5 | 400 |  | 100 |  | Belimumab |
| **SLE20** | 44 | f | 2 | 0 | 1:320 | 86.8 | neg | neg | neg | neg | neg | 939 |  | 4 | 200 | 2500 |  |  |  |
| **SLE21** | 30 | f | 4 | 0 | 1:160 | 47,3 | neg | neg | neg | neg | neg | 386 |  | 2.5 | 200 | 2000 |  |  |  |
| **SLE22** | 37 | f | 12 | 8 | 1:1280 | >200 | neg | pos | pos | pos | neg | 4349 | urticaria vasculitis | 5 | 300 | 1500 |  |  |  |
| **SLE23** | 28 | f | 4 | 0 | 1:640 | 84,6 | neg | neg | neg | neg | neg | 3543 |  | 5 | 200 |  |  |  |  |
| **SLE24** | 38 | f | 4 | 0 | 1:2560 | 102,6 | neg | pos | pos | neg | neg | 942 |  |  | 200 |  |  |  |  |
| **SLE25** | 45 | f | 0 | 0 | 1:640 | 8.4 | neg | pos | neg | neg | neg | 2375 |  |  | 200 |  |  |  |  |
| **SLE26** | 63 | f | 7 | 3 | 1:2560 | 173.3 | neg | pos | pos | neg | neg | 2153 | alopecia, thrombopenia |  | 200 |  |  |  |  |
| **SLE27** | 32 | f | 4 | 4 | 1:2560 | 10,3 | pos | pos | pos | pos | pos | 1733 | arthritis |  |  | 1000 |  |  | Belimumab |

## Supplementary Table 4. Staining panels applied for the CyTOF analysis.

| **Staining A** | | **Staining B** | |
| --- | --- | --- | --- |
| CD45 | Y89 | CD45 | Y89 |
| ICOS | Pr141 | ICOS | Pr141 |
| CD19 | Nd142 | CD19 | Nd142 |
| VISTA-908 | Nd143 |  |  |
| CD69 | Nd144 | CD69 | Nd144 |
| CD4 | Nd145 | CD4 | Nd145 |
| IgD | Nd146 | CD8 | Nd146 |
| CD11c | Sm147 | CD11c | Sm147 |
| CD274/PD-L1 | Nd148 |  |  |
| CD25 | Sm149 | CD25 | Sm149 |
| CD223/LAG-3 | Nd150 |  |  |
| CD123 | Eu151 | CD14 | Eu151 |
| TCRgd | Sm152 | CD21 | Sm152 |
| TIGIT | Eu153 | IgD | Eu153 |
| TIM3 | Sm154 |  |  |
| CD27 | Gd155 | CD27 | Gd155 |
| CD86/B7.2 | Gd156 | CD86 | Gd156 |
| CD137/4-1BB | Gd158 | CD10 | Gd158 |
| Foxp3 | Tb159 | FoxP3 | Tb159 |
| CD14 | Gd160 | IgG | Gd160 |
| CD152/CTLA-4 | Dy161 | IgA | Dy161 |
| CD8 | Dy162 |  |  |
| CD272/BTLA | Dy163 | BTLA | Dy163 |
| CXCR5 | Dy164 | CD23 | Dy164 |
| CD45R0 | Ho165 | CD45R0 | Ho165 |
| CD155 | Er166 | CD24 | Er166 |
| CD38 | Er167 | CD38 | Er167 |
| Ki-67 | Er168 | Ki-67 | Er168 |
| CD45RA | Tm169 | CD45RA | Tm169 |
| CD3 | Er170 | CD3 | Er170 |
| CD226 | Yb171 | CD20 | Yb171 |
| IgM | Yb172 | IgM | Yb172 |
| CD162/PSGL | Yb173 |  |  |
| HLA-DR | Yb174 | HLA-DR | Yb174 |
| CD279/PD-1 | Lu175 | CD71 | Lu175 |
| CD56 | Yb176 | CD56 | Yb176 |
| CD16 | Bi209 | CD16 | Bi209 |
