# Supplementary figures and images for: Deep Phenotyping of CD11c+ B Cells in Systemic Autoimmunity and Controls
Source: Front Immunol. 2021 Mar 12;12:635615. doi: 10.3389/fimmu.2021.635615 (PMC7994903; doi:10.3389/fimmu.2021.635615)

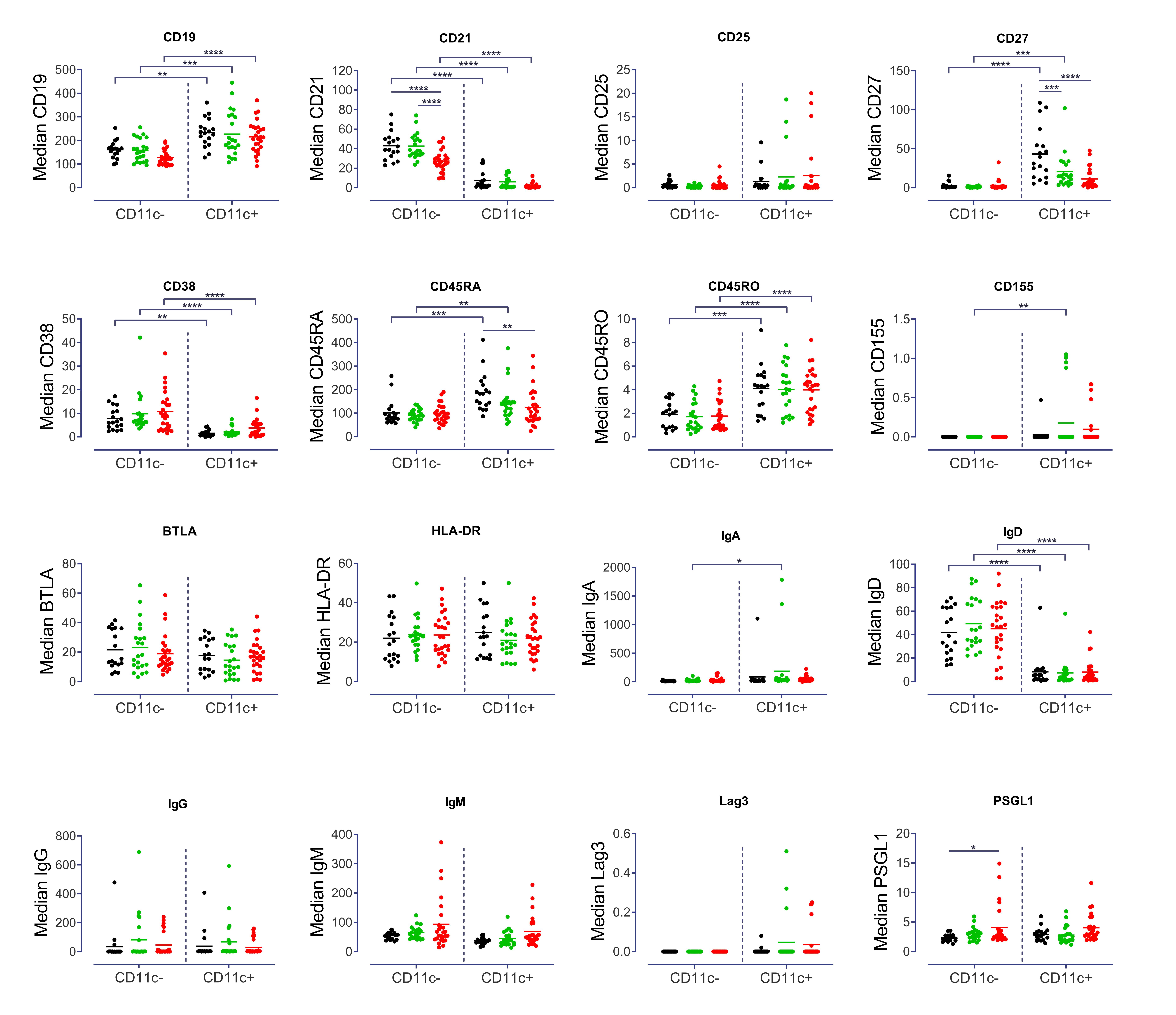

Supplement: Supplementary file 4 [file Image_3.jpg]
